# Genetic Markers Related to Meat Quality Properties in Fattened HF and HF x Charolaise Steers

**DOI:** 10.3390/genes15070843

**Published:** 2024-06-27

**Authors:** Piotr Kostusiak, Emilia Bagnicka, Beata Żelazowska, Magdalena Zalewska, Tomasz Sakowski, Jan Slósarz, Marcin Gołębiewski, Kamila Puppel

**Affiliations:** 1 Institute of Animal Science, Warsaw University of Life Sciences, Ciszewskiego 8, 02-786 Warsaw, Poland; piotr_kostusiak@sggw.edu.pl (P.K.); jan_slosarz@sggw.edu.pl (J.S.); marcin_golebiewski@sggw.edu.pl (M.G.); 2 Institute of Genetics and Animal Biotechnology, Polish Academy of Science, Jastrzębiec, Postępu 36A, 05-552 Magdalenka, Poland; e.bagnicka@igbzpan.pl (E.B.); b.zelazowska@igbzpan.pl (B.Ż.); t.sakowski@igbzpan.pl (T.S.); 3 Department of Bacterial Physiology, Institute of Microbiology, Faculty of Biology, University of Warsaw, 02-096 Warsaw, Poland

**Keywords:** beef, carcass quality, cattle, marker-assisted selection, meat trait

## Abstract

This study involved 45 Holstein and 60 Holstein-Charolaise steers, tailored with specific diets according to breed and rearing systems. DNA genotyping was conducted for DGAT1, LEP, SCD1, SREBF1, and TG genes to investigate their impact on carcass conformation traits, beef quality traits, and sensory quality traits. The results showed associations between the genetic variants and the analyzed traits. Specifically, DGAT1 was found to affect drip loss, meat brightness, and color saturation. The TG gene was associated with marbling and meat color. LEP influenced trim fat and pH levels, while SCD1 was linked to metabolic energy live weight gains, and pH levels. SREBF1 was related to fatness.

## 1. Introduction

The recent availability of genome sequencing methods and many previously identified molecular markers offer new opportunities for animal breeding, including the use of molecular information in selection programs [1]. Single nucleotide polymorphisms (SNPs) are one of the most common DNA variations in mammals—they are biallelic, abundant, and easy to detect. Even though beef production traits are influenced by polygenic regulation, it is essential to apply SNP-based genetic markers to obtain animals with better genetic backgrounds. Cattle with better genetics will develop desirable meat carcass conformation and quality and sensory properties. Although meat quality depends on animal genetics, it is also affected by environmental factors—animal feed, keeping conditions, welfare, etc. Ensuring proper environmental conditions for animals with high genetic potential may affect meat quality production yield. Animals with a proper genetic background will convert fodder more efficiently [2]. This might be important in countries with high milk production and the resulting large number of calves that could be used for more efficient and profitable fattening [3].

There have been many studies that aim to assess the effect of SNPs on beef quality; some of these are characterized by a broad range of applications, while others are specific only to small populations [4]. For the purposes of our study, we decided to use the five most extensively studied genes: Stearoyl-CoA desaturase (SCD), leptin (LEP), thyroglobulin (TG), diacylglycerol O-acyltransferase (DGAT1), and sterol regulatory element binding transcription factor (SREBF11). SCD is one of the main lipogenic enzymes in the fatty acid synthesis pathway in mammalian adipocytes and participates in the conversion of saturated fatty acids to unsaturated fatty acids; thus, the composition of stored fatty acids depends on SCD’s action [5,6]. Leptin’s main function is to regulate the assimilation, storage, and use of energy from nutrients [7]. TG regulates metabolism and fat deposition and also participates in fat cell development [8]. DGAT1 is engaged in triglyceride synthesis pathways and catalyzes the last step of the triglyceride’s synthesis from diacylglycerol to fatty acids [9]. SREBF11 is an important transcriptional activator for several lipogenic genes [10]. Issues related to marker-assisted selection implementation have been described by Zalewska et al. [4].

Consumers care about high nutritional quality [11], but at the same time, they are interested in favorable sensory attributes. In this study we analyze genetic polymorphism in the cattle breed that predominates in the milk belt of the northeastern part of Europe—the Holstein Friesian (HF). It is well known that the growth potential and carcass composition of HFs are generally worse compared to both dual-purpose and beef breeds [12,13,14]. However, such differences in growth rates, especially between HF and the early-maturing beef breeds, should be negligible [15]. The meat quality and sensory parameters as well as carcass composition are measured in male calves, which are culled mostly when they have a live body weight of 100 kg—but this may be too early. For study purposes, we determined the same parameters in HF and HF x Charolaise crossbred steers. Crossbreeding with Charolaise was introduced to improve beef quality.

Among the many studies exploring the influence of SNPs on beef quality, our research focuses on five extensively studied genes: SCD, LEP, TG, DGAT1, and SREBF11. These genes play pivotal roles in various metabolic pathways governing fatty acid synthesis, energy regulation, and lipid metabolism, thereby exerting profound effects on meat quality attributes. In this study, we investigate genetic polymorphisms within the HF cattle breed, prevalent in the milk belt of northeastern Europe. Despite HF’s historical association with suboptimal growth potential and carcass composition compared to beef breeds, efforts to enhance beef quality have been undertaken through crossbreeding with Charolaise cattle. Our research aims to clarify the impact of transitioning to high-quality feed and the utilization of select dietary supplements on product quality improvement, while also delineating the relationship between genetic factors and phenotypic traits. 

Our study is driven by two overarching objectives:

1. To assess the influence of transitioning to high-quality feed and dietary supplementation on product quality enhancement.
2. To determine the relationships between identified genetic factors and phenotypic traits associated with meat quality in HF and HF x Charolaise crossbred steers.

## 2. Material and Methods

### 2.1. Animals and Feeding Experiment

This study was conducted on 45 Holstein (HF) steers and 60 crossbred HF x Charolaise (HFxCH) steers. All the animals were castrated between three to four weeks old using the elastration method. In year 1, steers were kept on semi-natural pasture for the summer season and fed in a barn in winter. The animals were under the constant care of a veterinarian. In the performed 2 × 2 fattening experiment carried out on HF and crossbred HFxCH steers, HF steers were compared to HFxCH steers in a closed loop, fattened from weaning to slaughter at 15 and 18 months of age. Then, carcasses were chilled to 2–4 °C, and samples of semimembranosus muscle (300 g) were cut parallel to the muscle axis at 24 h postmortem. The animals were divided into four groups; all four groups were fed with grass silage with the addition of distillery spent grains and rapeseed meal. EUROP trade grades and fat classes were estimated by graders at the slaughterhouse. Carcasses were classified as having fat classes from 1 to 2+, while trade classes ranged from E to P. 

Trim fat was defined as the combination of subcutaneous and intermuscular fat deposits that could be discerned and separated using a standardized cutting procedure with a knife. This procedure ensured consistency and reproducibility in the assessment of fat content within the carcass. Subcutaneous fat refers to adipose tissue located beneath the skin, while intermuscular fat resides between muscle bundles.

All steers had unlimited access to pasture for 1 year of their life. Forage availability was determined monthly using a calibrated plate meter. Forage height was measured in a 0.210-m^2^ quadrat using a rising plate meter before being hand-clipped to ground level. Forage samples were collected by hand at random locations within each pasture, representative of animal diet, for determination of forage quality. The pasture samples were composed of approximately 63.45% Organic Matter Digestibility (OMD), 16.21% Crude Protein (CP), 43.54% Acid Detergent Fiber (ADF), 54.67% Neutral Detergent Fiber (NDF), and 10.08% Ash (A). The pasture was offered to the steers at 4% of Live weight (LW).

After the grazing period, animals were moved to a feeding unit where they were kept for a finishing phase to assess the impact on their yield and meat quality. Steers fed for 15 months (=3 months of intensive feeding) are considered as “low intensity” and those fattened for 18 months as “high intensity”. The finishing ratios fed at this stage are displayed in Table 1. During this phase, the steers were offered two ratios: intensive and semi-intensive, followed by 2 transitions diets. Rations 1 and 2 were step-up diets and were provided for 7 d each before finishing diets (3 and 4) were introduced.

All steers had unlimited access to pasture in 1 year of their life, which means that all steers were fed with the same diet. After the grazing phase animal were divided into two feeding groups (semi-intensive and intensive (Table 1; rations 3 and 4). To get the animals used to a changed diet, two step-up diets (Table 1; diets 1 and 2) were introduced. Diet 1 preceded diet 3 and diet 2 preceded diet 4. To summarize, there were two genetic groups (HF and HFxCH) × two feeding groups (fed with diets 3 and 4).

### 2.2. Slaughter, Carcass and Meat Quality

#### 2.2.1. Weight Gains

The weighing of the steers was carried out using the CalmScale system (Jantar Sp. z o.o., Bielsko-Biała, Poland), which aims to minimize stress. The system, installed in the cattle’s watering area, utilizes RFID tags and antennas for precise identification. Weight data, along with other pertinent details, were recorded for analysis multiple times each day as the animals drew water. The average daily gain (ADG) was calculated by subtracting the initial weight from the current weight and dividing it by the number of days since the initial measurement, thus providing a key metric for evaluating steer growth.

#### 2.2.2. Color Assessment

Color measurements were performed in the CIE L*a*b* system using a Minolta CM 2022 (Konica Minolta, Tokyo, Japan). The procedure of color determination included sampling a slice of meat (ca. 2 cm thick) at 3 points (results obtained were averaged). Hue (b*/a*) and chromaticity (√(a*^2^ + b*^2^)) of meat sample color were calculated according to the formula provided by Mordenti et al. [16].

Meat Color (Brown and Bright): Meat coloration is influenced by various factors including myoglobin content, pH level, and cooking methods. “Brown meat color” typically indicates the development of Maillard reaction products and denatured proteins, resulting in a darker appearance, while “bright meat color” suggests a lighter or more vibrant hue, possibly indicative of less cooking or lower myoglobin content.

#### 2.2.3. Marbling Scores and Yield Grades

Marbling scores and yield grades were directly estimated using the VIA-based camera system (VBG 2000) [17] at the rib-eye cut between the 10th and 11th rib interfaces of the longissimus thoracis. Additionally, all halves were further ribbed to assess marbling scores and yield grades, which encompassed factors such as meat surface, rib fat thickness, and carcass weight.

#### 2.2.4. Taste Characteristic

An electronic tongue system was used to determine the taste characteristics of the muscle tissue. The system was composed of five taste sensors, with each sensor being attached to a typical artificial lipid membrane. The sensors were named CA0 (to detect sour substances), C00 (to detect bitter substances), AE1 (to detect astringent substances), AAE (to detect umami substances), and CT0 (to detect salty substances). All the sensors were pre-conditioned in a reference solution for one day before the measurements were taken.

#### 2.2.5. Drip Loss

Drip loss was determined using samples of homogenized meat weighing 40 g, which were tightly packed into glass weighing dishes. The samples were then submerged and kept at 70 °C for 15 min in a heated bath. After that time, the meat samples were removed from the weighing dishes and left for 24 h to allow the water to drip out. The difference in weight was then measured and expressed as a percentage of the original sample weight (51).

#### 2.2.6. pH

The meat’s pH was determined using a pH meter HI 99163 with a temperature measurement function and a probe tip ending in a stainless-steel knife, facilitating measurements (Hanna Instruments, Providence, RI, USA).

#### 2.2.7. Shear Force

The carcasses were chilled to 2–4 °C and samples of semimembranosus muscle (300 g) were cut parallel to the muscle axis at 24 h postmortem, after which muscle sections measuring 2 × 2 × 2 cm were then cut from the samples. Shear force was determined using the Zwick 5.0 Zwicki—Line strength testing machine (Zwick Roell Polska Sp. z o. o. Sp. k., Wrocław, Poland) [18].

#### 2.2.8. Carcass Conformation and Fat Cover

Carcass conformation and fat cover were evaluated in accordance with the European Union Carcass Classification Scheme (EUROP; Council of the European Union, No 1234/Citation2007; Commission of the European Union, No 1249/Citation2008). Conformation assessment focused on the development of specific anatomical regions, including the round, back, and shoulder, with consideration given to their muscularity and overall shape. The EUROP classification system assigns grades denoted as follows: E (excellent), U (very good), R (good), O (fair), P (poor). The fat cover assessment involved the examination of subcutaneous fat and intrathoracic fat deposits. This evaluation utilized a numerical scale ranging from 1 to 5, with each grade indicating varying levels of fat deposition: 1: low; 2: slight; 3: average; 4: high; 5: very high.

Dry Matter Live Weight Gain: This parameter signifies the increase in an animal’s body weight excluding water content. It quantifies the net accumulation of structural and functional components such as proteins, fats, and minerals, which are essential for growth and development. Metabolizable Energy Live Weight Gain: This represents the increment in an animal’s weight attributable to the assimilation and utilization of metabolizable energy derived from its diet. Metabolizable energy refers to the portion of dietary energy that is available for physiological processes such as maintenance, growth, and production after accounting for losses due to digestion and metabolism. 

Live Weight Gain (LWG) was calculated by subtracting the initial live weight from the final live weight of the animals over the specified period. Dry Matter Live Weight Gain (DM_LWG) was determined by multiplying the LWG by the dry matter percentage of the gained weight. Metabolic Energy Live Weight Gain (ME_LWG) was calculated based on the metabolizable energy content of the gained weight. These calculations provided crucial insights into the growth performance and energy utilization efficiency of the animals during the study period.

### 2.3. DNA Sampling and Analysis

#### DNA Analysis

Blood samples were collected in tubes with anticoagulating agents from fattened HF (n = 45) and HF x Charolaise (n = 60) crossbred steers before slaughter at 15 and 18 months of age. Total DNA from whole blood was isolated using DNeasy Blood & Tissue (Qiagen, Hilden, Germany) according to the manufacturer’s protocol. The quantity of the extracted DNA was analyzed using an Invitrogen Qubit 4.0 Fluorometer (Thermo Fisher Scientific, Waltham, MA, USA) with a dsDNA high-sensitivity assay kit; and the quality was determined using a NanoDrop 2000 spectrophotometer (Thermo Fisher Scientific, Waltham, MA, USA). The DNA samples were isolated in triplicate and then pooled for each animal. 

DNA isolation and genotyping were performed at the Institute of Genetics and Animal Biotechnology PAS, Jastrzębiec, Poland, using the RFLP method. The following genes were chosen for analysis: Stearoyl-CoA desaturase (Δ-9-desaturase) (SCD1), leptin (LEP), thyroglobulin (TG), diacylglycerol O-acyltransferase (DGAT1), and sterol regulatory element binding transcription factor (SREBF11), as they are all linked to the assessment of qualitative beef traits. Genes, primer sequences restriction enzymes, and polymorphisms are presented in Table 2. PCR was conducted using AmpliTaq Gold DNA Master Mix (Thermo Fisher, Waltham, MA, USA) and PCR conditions were optimized for each reaction according to the polymerase manufacturer’s protocol.

The presence of the genes of interest after the PCR was confirmed by electrophoresis in a 1.5% agarose gel (55 V, 50 min). Restriction enzyme digestion (Supplement Table S1) was conducted according to the manufacturer’s recommendations for each digestion enzyme. The presence of bands of interest after digestion was confirmed using electrophoresis in a 2–3% agarose gel (55 V, 50 min), depending on the size of the band of interest.

Phenotypic data on daily meat yield and composition were obtained from the meat control system. This phenotypic data contained information on the quality and sensory parameters of selected traits for each animal’s breed; date of birth; date of slaughter; daily gains; carcass weight; slaughter yield; evaluation of the quality of muscle and fatness of the carcass; marbling; pH after slaughter; carcass temperature after slaughter; pH after 48 h; carcass temperature at 48 h after slaughter; thawing loss due to carcass defrosting as, a percentage, after 7 and 14 days; cooking losses, as a percentage, after 7 and 14 days; pH 7 and pH 14; meat color as L *, a *, b * after 7 and 14 days; meat color in terms of lightness or darkness; appearance of fat cover; texture; juiciness; and palatability in terms of sweet, sour, and metallic taste.

### 2.4. Statistical Analysis

An analysis of variance was performed using the GLM procedure in an SAS package (SAS software, version 9.2; Statistical Analysis System Institute Inc., Cary, NC, USA) [21] to determine the significance level of all identified factors that may have affected the tested traits or influenced the relationship between the analyzed traits and the specific genotypes. All identified factors had a significant impact on the examined traits; therefore, they were included in the final model. Body weight and age at the start of the experiment and at the end of the experiment were treated as linear regressions in the model (body weight on the day at the start of fattening, age of the animal on the day at the start of fattening, age of the animal on the day of slaughter, weight of the animal on the day of slaughter).

y_ijklmno_ = µ + BREED_i_ + FATTENING_j_ + BREED*FATTENING_ij_ + β_o_ (LWSTART_k_) + β_o_ (AGESTART_l_) + β_o_ (LWSLAUG_m_) + β_o_ (AGESLAUG_n_) + e_ijklmno_ (1)

where:

y_ijklmno_-investigated trait

µ-overall mean

BREED_i_-fixed effect of i-th steer breed (HF, HF x CH)

FATTENIN_j_-fixed effect of j-th fattening type (intensive or extensive)

BREED*FATTENING_ij_-interaction between i-th BREED and j-th type of fattening

β_o_ (LWSTART_k_)-linear regression on the body weight on the day feeding was started

β_o_ (AGESTART_l_)-linear regression on the age on the day feeding was started

β_o_ (LWSLAUG_m_)-linear regression on the body weight on the day of slaughter

β_o_ (AGESLAUG_n_)-linear regression on the age on the day of slaughter

e_ijklmno_-random error

Factors that did not significantly influence the investigated meat and carcass quality traits were removed from the presented model.

The databases used in the analysis compiled the genotypes of individual genes for all animals along with the following information about the animals: breed, date of birth, date of slaughter, and information about their productivity and meat quality taken at the time of slaughter. Prior to the statistical analysis, we sorted the parameters into four groups and analyzed the relationships between genotypes for selected genes and groups separately.

The first group included parameters concerning the carcass conformation traits of HF and HFxCH steers (fatness, marbling, trim fat, live weight gain [LWG], dry matter live weight gain [DM_LWG], and metabolic energy live weight gain [ME_LWG]). The second group consisted of factors describing the beef quality traits of HF and HFxCH steers (drip loss as a percentage after 7 days [Withaw7]; thermal drip loss as a percentage after 7 days [Wlcook7]; pH after 7 days [pH 7]; meat brightness after 7 days [L7]; meat red-color saturation after 7 days [a7]; meat yellow-color saturation after 7 days [b7]; drip loss as a percentage after 14 days [Withaw14]; thermal drip loss as a percentage after 14 days [Wlcook14]; pH after 14 days [pH 14]; meat brightness after 14 days [L14]; meat red-color saturation after 14 days [a14]; meat yellow-color saturation after 14 days [b14]; age at slaughter). The latter group of analyzed traits related to the quality of beef sensory traits of HF and crossbred steers (the meat’s brown color, the meat’s brightness, shear force, juiciness, tenderness, and taste–total meat taste and sweet, sour, umami and metallic taste). The phenotypic data covered information on the quality and sensory characteristics of the assessed traits and were determined 24 h after slaughter (or as stated otherwise). 

## 3. Results

### 3.1. Allele Frequencies

In the analysis of SCD1, digestion was accomplished using an Fnu4HI (SatI) enzyme, which recognizes the CC/NGC sequence. The analyzed mutation is a T>C replacement, resulting in a non-synonymous mutation that leads to a change of the valine amino acid into alanine in the protein chain. The genotype frequencies were as follows: VV = 5.7%, AA = 55.0%, and VA = 39.3%. In the analysis of the LEP polymorphism, the digestion was performed by the enzyme BspEI, which recognizes the T/CCGGA sequence. This mutation is a T>C replacement. the genotype frequencies were as follows: CC = 23.2%, TT = 19.1%, and CT = 57.7%. In terms of TG, the restriction enzyme BstYI, which recognizes the RGATCY sequence, was used to determine the SNPs’ variants, and the sought mutation is a T>C replacement. The genotype frequencies were CC = 73.5%, CT = 23.5%, and TT = 3.0%. For DGAT1, digestion was performed by the enzyme CfrI, which recognizes the Y/GGCCR sequence. The mutation sought is a T>C replacement and the genotype frequencies were as follows: CC = 83.9% TT = 8.8%, and CT = 7.3%. The mutation in the analyzed fragment of SREBF11 consisted of the deletion of 84 bp. Genotype frequencies were as follows: no deletion = 91.1% and heterozygotes = 8.9% (Table 3).

### 3.2. The Relationship between the Selected Traits and the Analyzed SNPs

During the study we found fatness to be associated with cattle breed, with higher values for the crossbreed (*p* ≤ 0.01); and the SREBP11 LL homozygote (*p* ≤ 0.05) genotype; however, we did not find the SS homozygote in the tested population. We also determined fatness to be associated with breed and intensity as a combined effect (Table 4). In terms of marbling, we found higher values for the crossbreed (*p* ≤ 0.01), while for TG we found higher values for TT than for CT (*p* ≤ 0.05) and CC (*p* ≤ 0.05). Also, rearing intensity affected marbling with higher values for ‘low’ (*p* ≤ 0.01). Moreover, we demonstrated marbling to be associated with breed and intensity as a mixed effect (Table 4). As for trim fat, we found higher values for the crossbreed (*p* ≤ 0.05), while for *LEP* we found higher values for the CC homozygote than for the TT homozygote (*p* ≤ 0.05). During the study, we identified that LWG was associated with the breed (higher values for the crossbreed, *p* ≤ 0.01), rearing intensity (higher values for ‘high’, *p* ≤ 0.01), and breed–intensity as a combined effect (Table 4), with the analyzed genes’ genotypes having no effect on this parameter. As for DM_LWG, we found it to be linked to rearing intensity, with higher values for ‘low’ (*p* ≤ 0.01), and with breed and intensity as a combined effect (Table 4). No effect was found for the analyzed genes’ genotypes on this parameter. For ME_LWG we found associations with breed and intensity as a combined effect, and with the SCD1 genotype, with lower values for AA than for VA (*p* ≤ 0.05) and VV (*p* ≤ 0.05). We found that DGAT1 did not have any effect on the analyzed carcass conformation traits. All the above-mentioned associations are presented in detail in Table 4.

We found that the polymorphic forms of the chosen genes affected beef quality traits. Wlthaw7 was associated with the breed (with higher values for the crossbreed, *p* ≤ 0.05), and the DGAT1 polymorphism (higher values for TT than CC homozygote, *p* ≤ 0.05). We found that Wlcook7 was related to the LEP polymorphism (with higher values for TT than for CC homozygote, *p* ≤ 0.05). The pH 7 parameter was associated with rearing intensity (with higher values for ‘low’ type, *p* ≤ 0.05) and the SCD1 genotype (higher values for AA than VA, *p* ≤ 0.01, and VV, *p* ≤ 0.05). We identified that L7 related to breed (higher values for the crossbreed, *p* ≤ 0.01), the DGAT1 genotype (the highest value for heterozygote), the TG genotype (higher values for CC than heterozygote, *p* ≤ 0.05), and the SCD1 genotype (higher values for heterozygote than CC homozygote, *p* ≤ 0.05). We found that a7 was related to breed (higher values for the crossbreed, *p* ≤ 0.01), the TG polymorphism (higher values for TT than heterozygote, *p* ≤ 0.01), and the LEP genotype (higher values for the heterozygote than CC genotype, *p* ≤ 0.05); while b7 was related to breed (higher values for the crossbreed, *p* ≤ 0.01) and the TG polymorphism (higher values for the CC genotype than for heterozygote, *p* ≤ 0.01). We determined that Wlthaw14 was associated with rearing intensity (higher values for “low”, *p* ≤ 0.01) and with the TG genotype (higher values for CC than TT homozygote, *p* ≤ 0.01). Wlcook14 changed with the SCD1 polymorphism (higher values for heterozygote, *p* ≤ 0.05). We found that pH 14 was associated with LEP (the highest value for the CC genotype) and with the SCD1 polymorphisms (higher values for heterozygote than AA homozygote, *p* ≤ 0.05). We identified that L14 related to breed (higher values for the crossbreed, *p* ≤ 0.01), the DGAT1 polymorphism (the lowest value for CC homozygote), and the SCD1 polymorphism (higher values for heterozygote compared with AA homozygote, *p* ≤ 0.05). We also found associations between a14 and breed (higher values for the crossbreed, *p* ≤ 0.05), rearing intensity (higher value for low type, *p* ≤ 0.05), and the DGAT1 polymorphism (higher value for TT homozygote than for CC, *p* ≤ 0.05); while b14 was related to breed (higher values for the crossbreed, *p* ≤ 0.01) and the DGAT1 polymorphism (higher values for TT homozygote than for CC, *p* ≤ 0.05). We did not detect any effect of the SREBF11 polymorphism on the analyzed beef quality traits. All the abovementioned associations are presented in detail in Table 5.

Moreover, we identified the analyzed parameters that affected beef quality traits. The brown meat color was related to the breed (with higher values for dairy, *p* ≤ 0.05), and the bright meat color was also associated with the breed (with a higher value for dairy, *p* ≤ 0.01). Shear force was only associated with the breed (with a higher value for crossbreed, *p* ≤ 0.05). Of the analyzed SNPs, we found that the only polymorphism to affect the sweet taste was SCD1, with higher values for heterozygote, than VV homozygote, *p* ≤ 0.05. The DGAT1, TG, LEP, and SREBF11 polymorphisms did not affect any of the analyzed beef sensory quality traits. All the above-mentioned associations are presented in detail in Supplement Table S2.

## 4. Discussion

The molecular analysis allowed for the identification of potential candidate genes that may control economically important traits in beef production. Applying genomic techniques to breeding programs helps achieve progress faster than classic breeding methods. During the study, we focused on genetic markers related to meat quality properties in fattened HF and HF x Charolaise steers. We analyzed the effect of breed, rearing intensity, and selected polymorphisms in LEP, DGAT1, SCD1, SREBF11, and TG on carcass conformation traits, beef quality traits, and beef sensory quality traits.

It is difficult to discuss these results only in terms of the selected SNPs’ effect on measured traits because all traits determined in this study depended greatly on the cattle breed. The polymorphisms that affect carcass conformation, or its quality and sensory properties are in most cases analyzed in fast-growing beef cattle or, alternatively, in dual-purpose cattle, and the various crossbreeds between them. The most extensively studied breeds belong to Angus, Hereford, Limousin, Charolaise, Gelbvieh (historically triple purpose, nowadays mainly for beef), some local beef breeds such as Chinese Qinchuan, Canchim or Caracu cattle, or dual-purpose cattle such as Simmental or Valdostana [22,23,24]. Nkrumah et al. stated that lines based on Angus and Hereford had, in general, higher carcass and body fat, and lower carcass leanness compared to lines based on Gelbvieh, Limousin, or Charolaise; such discrepancies in studied herds may generate more complications in interpreting the selected polymorphisms’ associations with the traits [24]. The genetic background of the studied animals is also important because sometimes local breeds are not classified as pure *Bos taurus*, they may have been mated with *Bos indicus* cattle. Lin et al. have studied the genetic distance between *B. taurus* and *B. indicus* and found that the genetic distances between Asian and European cattle populations are generally high, with the greatest level of genetic differentiation detected between *B. taurus* and *B. indicus* populations (any population differentiation was not observed among any pairs of populations by exact tests) [25]. Additionally, it is difficult to find studies applied to dairy cattle, which is the most widespread breed across Europe: with approximately 20.5 mln animals in 2021, while non-dairy cattle reached only 10.5 mln [26].

### 4.1. LEP

Leptin is considered to be associated with carcass quality traits (fat, animal body weight, growth rate) due to its main function [7]. During our study, we focused on SNPs that were described initially by Buchanan et al. and found Leptin to be associated with carcass conformation (trim fat) and beef quality (Wlcook7, a7, pH 14). Any effect of *LEP* polymorphism on beef sensory traits was not found [19]. The results from a study performed by Nkrumah et al. partially agreed with our observations, i.e., researchers did not find any associations between the analyzed SNP and carcass marbling for animals with different genotypes [24]. However, they did find that animals with TT genotypes had more carcass fat grade than those with CC genotypes, in contrast to our results—we found that animals with CC genotypes had more trim fat than TT homozygotes. In a similar way to our results, other researchers did not find any association between the LEP polymorphism and marbling [22,23], tenderness [23], or carcass fat thickness [22] found changes only at the trend level within their studied population, with the highest value being for TT). Moreover, Carvalho et al. found the shear force to be related to the LEP polymorphism, with higher values for the CC than the TT genotype, and the TT than the CC genotype, *p* ≤ 0.05 [22]. Due to the primary role of leptin as a regulator of appetite, body weight gain, and fat deposition [27], LEP polymorphism was considered regarding carcass conformation traits (e.g., live weight, live weight gain, marbling score, fatness); thus we attempted to assess its value also in relation to quality traits such as meat color, pH, and drip loss, together with beef sensory traits. We did not find any associations between the LEP polymorphism and beef sensory traits; however, we did find that it influenced Wlcook7, a7, and pH 14, which has not been reported previously.

### 4.2. SCD1

Stearoyl-CoA desaturase 1 (SCD1) is an integral membrane protein that is involved in the biosynthesis of monounsaturated fatty acids from saturated fatty acids. SCD1 catalyzes the insertion of a *cis* double bond at the delta-9 position of a range of acyl-CoA substrates, including palmitoyl-CoA and stearoyl-CoA1. This enzymatic activity is crucial for the formation of oleic and palmitoleic acids, which are major components of membrane phospholipids, triglycerides, and cholesterol esters. In this study, we found SCD1 to be associated with carcass conformation (metabolic energy live weight gains), beef quality (pH 7, pH 14, Wlcoock14, L7, L14), and beef sensory traits (sweet taste). The results demonstrated the effect of AA, Va, and VV forms on pH 7; and AA, VA forms on pH 14. These results do not match those of Li et al. [28]; however, SCD1 could indirectly influence pH levels due to changes in metabolic processes [10]. While the specific influence of SCD1 on beef lightness of color or brightness is not widely documented, it is plausible that variations in fat composition, influenced by SCD1, could indirectly affect these visual aspects of meat quality. The brightness or lightness of meat color is often associated with fat content and marbling [29] or potentially muscle fiber diameter as a result of pH changes [30]. Our results show that SCD1 is associated with L7 and L14 for AA and VA genotypes. These results are partially confirmed by Reardon et al. who showed that the AA genotype had a significant effect. However, these results need to be verified in future studies [31]. 

The relationship between Stearoyl-CoA Desaturase 1 (SCD1) and thermal drip loss in beef has not explicitly been addressed in the literature yet. The results presented in our study point to the AA and VA genotypes as being potentially important in shaping the thermal drip loss trait. Various studies have investigated the genetic factors influencing drip loss, primarily in pork, which may provide indirect insights relevant to beef and potentially to SCD1. For example, Li et al. identified the triadin (TRDN) and myostatin (MSTN) genes as critical candidates for drip loss in pork due to their roles in muscle contraction and growth [32]. Ponsuksili et al. identified differentially regulated transcripts in pork muscle that were related to membrane proteins, signal transduction, and lipid metabolism, which affected water-holding capacity and drip loss [33]. Reardon et al. indicated a similar relationship between PRKAG3 and thermal drip loss in beef as well as in pork [31]. Considering SCD1′s role in lipid metabolism, it might indirectly influence drip loss in beef through its effects on muscle fat composition and membrane properties. However, since current research has not directly established a link between SCD1 and thermal drip loss, it remains a speculative candidate. Further studies specifically investigating the role of SCD1 in relation to meat quality traits, including thermal drip loss, would be necessary to clarify its potential.

The expression of SCD1 in cattle is linked to crucial traits such as intramuscular fat content or marbling, a primary determinant of meat quality affecting flavor, tenderness, and juiciness. Studies by Ardıçlı et al. have demonstrated an association between SCD1 gene polymorphisms and variations in fat deposition and marbling in cattle breeds, directly influencing the quality of beef where the A allele was associated with higher performance than the G allele [34]. Additionally, SCD1 activity has implications for feed efficiency and weight gain in cattle, indicating that genetic variations in this gene can affect how efficiently cattle convert feed into body mass. This is in agreement with our results. However, we found significant ME_LWG results in AA, VA, and VV genotypes. 

Among the analyzed SNPs, we found that the only polymorphism affecting sweet taste was SCD1, with higher values observed in heterozygotes compared to VV homozygotes, *p* ≤ 0.05. However, the DGAT1, TG, LEP, and SREBF11 polymorphisms did not influence any of the analyzed beef sensory quality traits.

### 4.3. TG

Thyroglobulin (TG) in cattle, which is essential for thyroid hormone synthesis, is crucial for regulating metabolism and fat deposition. Key polymorphisms in the TG gene significantly influence beef quality traits such as marbling and fat deposition. The TT genotype is often linked to higher marbling compared to the CC and CT genotypes [35,36], which is in agreement with our results. These genetic variations are utilized to improve beef quality. The TG gene, which affects carcass fat accumulation, directly influences intramuscular fat, which is vital for meat marbling and palatability. Despite study variations, TG polymorphisms seem to be important for cattle breeding aimed at enhancing meat quality [4]. 

Another important aspect of beef quality influenced by the TG gene is meat color, which includes traits like brightness, and red and yellow color saturation. The color of beef is an important factor affecting consumer perception. Studies have shown that specific TG gene polymorphisms can affect the saturation of red and yellow colors in beef. The variation in these color traits can be indicative of the meat’s freshness, processing, and overall quality. Ardicli et al. identified greater brightness associated with CC and higher red color saturation associated with CT [37]. Except for the yellow color saturation, the results of the Ardicli et al. research seem to partially match the results of our study [37]. However, it is necessary to carry out more research work concerning this case in the future.

Drip loss associated with CC and TT genotypes presented in this study seems to partially match Du et al.’s results [38]. Water holding capacity (WHC) is positively correlated with intramuscular fat content in muscles, which is determined by TG [39,40,41]. These studies, while not directly studying TG gene polymorphisms and their effect on drip loss or water loss in beef, provide a broader context for understanding the genetic factors that may influence these traits in beef. Further studies on the TG gene specifically focusing on these aspects of beef quality are needed to provide more direct conclusions.

### 4.4. DGAT1

DGAT1 is a gene that plays a crucial role in the synthesis of triglycerides, which are the main components of intramuscular fat (IMF). DGAT1 has been studied in relation to its association with beef production traits, and some studies have shown that DGAT1 has a positive effect on meat quality and carcass fatness [4]. DGAT1 has also been associated with marbling, which is an important factor in determining beef quality. It is an important candidate gene in the production of high-quality beef Yuan 2013 [9]. In this study, we identified that DGAT1 influenced drip loss after 7 days, brightness, and red and yellow color saturation. Xin Li et al. [28] in a similar study did not identify this effect; however, they found an effect on marbling which was not found in our study. The L7 and L14 effect was related to CC, CT, and TT, and a14 and b14 in CC and TT genotypes. Research by Ardicli et al. on Holstein cattle also found that DGAT1 had no effect on color or drip loss, but they suggest that it may affect color due to its influence on marbling [37]. The importance of DGAT1′s effect on meat quality and the limited literature available, indicate the need for more research in this area [42].

### 4.5. SREBF1

Sterol regulatory element-binding factor 1 (SREBF1) is a transcription factor that plays an important role in lipid metabolism and fat deposition in cattle. SREBF1 is involved in the regulation of genes associated with fatty acid synthesis and cholesterol metabolism. The mRNA synthesis of SREBF1 is regulated by nutrients, and its metabolic activity might be potentiated by diet components and changes in lipogenesis in muscle. Several studies have found correlations between the marbling score and meat flavor. Thus, nutrition and management strategies that are able to increase the intramuscular fat content might contribute to increasing the added value of beef [43,44]. Our results confirmed its impact on fatness via the LL and LS genotypes. Moreover, Maciel et al. reported that vitamin A supplementation might increase the expression of SREBF1 at weaning, which increases the chances of improving the quality of beef [45]. On the other hand, Berrios et al. note the possible negative effects of the T allele, which, in his/her study may have been responsible for conceptus death in Holstein cows on day 16 of pregnancy. Further research is needed to thoroughly analyze these risks [46].

## 5. Conclusions

In conclusion, our study revealed significant associations between various genetic polymorphisms and beef quality traits in cattle. Notably, we observed distinct effects of genetic variants on carcass conformation, fatness, marbling, and other sensory attributes of beef.

Specifically, we found that certain genotypes of genes such as SCD1, LEP, TG, and DGAT1 were associated with alterations in carcass composition and quality traits. For instance, the SCD1 AA genotype demonstrated a significant influence on parameters associated with fatness and meat tenderness compared to VA and VV genotypes. Similarly, the DGAT1 TT genotype exhibited correlations with variations in fat deposition and beef tenderness, particularly in relation to marbling and shear force, compared to CC and CT genotypes. Furthermore, our investigation uncovered relationships between genetic variations and sensory attributes of beef, encompassing meat color, tenderness, and taste perception. Notably, the SCD1 polymorphism emerged as a determinant of the sweet taste of beef, with heterozygous genotypes showing higher taste scores compared to VV homozygotes.

## Figures and Tables

**Table 1:** Ingredients and chemical composition of transition and finishing rations fed in the finishing stage.

Item	Ration			
Ingredient composition, % as-fed	1	2	3	4
Grass silage	76.8	69.1	59.3	53.5
Rapeseed meal	3.5	4.2	7.7	8.4
Distillers grains	10	15	20	22.3
Grain mix (triticale and barley (50:50)	9	11	12.3	15.1
Minerals	0.7	0.7	0.7	0.7
Chemical composition:				
DM, %	61.2	69.8	75.2	79.1
CP, %	14.2	15.5	16.6	17.5
Fat, %	3.2	3.8	4.3	4.9
NEm, Mcal/kg DM	1.7	1.9	2.1	2.3
NEg, Mcal/kg DM	1.2	1.3	1.5	1.6

Rations 1 and 2 = step-up diets fed for 7 d each; rations 3 and 4 = finishing diet.

**Table 2:** Genes, primer sequences, restriction enzymes, and genes polymorphisms.

Gene	Primers (F/R)	Amplicon Size [bp]	Restriction Enzyme	Polymorphism [bp]	Reference
SCD1	ATG TAT GGA TAC CGC CCT TAT	145	Fnu4HI	T>C replacement AA 29, 48, 68 bp VA 29, 48, 68, 116 bp VV 29, 116 bp	[10]
	TTC TGG CAC GTA ACC TAT ACC CT				
LEP	ATG CGT GTG GAC CCC TGT ATC	94	BspEI	T>C replacement CC 75 bp CT 75, 94 bp TT 94 bp	[19]
	TGG TGT CAT CCT GGA CCT CC				
TG	GGG GAT GAC TAC GAG TAT GAC TG	548	BstYI	T>C replacement CC 75, 178, 295 bp CT 75, 178, 295, 473 bp TT 75, 473 bp	[20]
	GTG AAA ATC TTG TGG AGG CTG TA				
DGAT1	GCA CCA TCC TCT TCC TCA AG	411	CfrI	T>C replacement CC 411 bp CT 203, 411 bp TT 203 bp	[20]
	GGA AGC GCT TTC GGA TG				
SREBF11	CCA CAA CGC CAT CGA GAA ACG CTAC	432	-	deletion of 84 bp LL* 432 bp LS 348, 432 bp SS* 348 bp	[10]
	GGC CTT CCC TGA CCN CCC AAC TTAG				

SCD1-Stearoyl-CoA desaturase, LEP-leptin, TG-thyroglobulin, DGAT1-diacylglycerol O-acyltransferase, SREBF11-sterol regulatory element binding transcription factor; *L-long, S-short.

**Table 3:** Allele Frequencies.

Gene	Enzyme	Recognition Sequence	Mutation	Genotype Frequencies
SCD1	Fnu4HI	CC/NGC	T>C	VV = 5.7%, VA = 39.3%, AA = 55.0%
LEP	BspEI	T/CCGGA	T>C	TT = 19.1%, CT = 57.7%, CC = 23.2%
TG	BstYI	RGATCY	T>C	TT = 3.0%, CT = 23.5%, CC = 73.5%
DGAT1	CfrI	Y/GGCCR	T>C	TT = 8.8%, CT = 7.3%, CC = 83.9%
SREBF11	N/A	N/A	84 bp deletion	No deletion = 91.1%, Heterozygotes = 8.9%

LEP-leptin, DGAT1-diacylglycerol O-acyltransferase; SCD1-Stearoyl-CoA desaturase; SREBF11-sterol regulatory element binding transcription factor; TG-thyroglobulin; N/A-Not Applicable.

**Table 4:** Effect of breed, rearing intensity, fattening type, and chosen gene polymorphisms on carcass conformation traits for HF and HFxCH steers.

Effect		FATNESS ^1^		MARBLING ^2^		TRIM FAT ^3^		LWG ^4^		DM_LWG ^5^		ME_LWG ^6^	
		LSM	Se	LSM	Se	LSM	Se	LSM	Se	LSM	Se	LSM	Se
**Breed**	1-dairy	7.87 ^A^	0.24	1.37 ^A^	0.16	9.28 ^a^	0.87	1.33 ^A^	0.03	6.4	0.17	76.32	2.04
	2-cross	9.41 ^A^	0.25	2.08 ^A^	0.16	12.47 ^a^	0.91	1.48 ^A^	0.03	5.98	0.17	71.21	2.1
**Intensity**	1-low	8.92	0.23	1.96 ^B^	0.15	10.64	0.87	1.34 ^B^	0.03	6.58 ^A^	0.16	76.44	2.01
	2-high	8.37	0.25	1.49 ^B^	0.17	11.11	0.92	1.47 ^B^	0.03	5.79 ^A^	0.17	71.09	2.14
**Breed×Intensity**	1 × 1	7.99 ^aB^	0.29	1.56 ^C^	0.18	8.48 ^bc^	1.13	1.27 ^CDE^	0.03	6.80 ^aB^	0.21	79.13 ^a^	2.61
	1 × 2	7.76 ^Cb^	0.31	1.19 ^DE^	0.2	10.09	1.19	1.38 ^CF^	0.03	5.99 ^a^	0.22	73.51	2.75
	2 × 1	9.85 ^BC^	0.31	2.36 ^DC^	0.2	12.80 ^b^	1.18	1.41 ^DG^	0.03	6.35 ^b^	0.22	73.75	2.74
	2 × 2	8.98 ^abd^	0.31	1.80 ^Ea^	0.2	12.13 ^c^	1.19	1.56 ^EFG^	0.03	5.60 ^Bb^	0.22	68.66 ^a^	2.75
**DGAT1**	1-CC	8.94	0.14	1.96	0.08	10.66	0.55	1.4	0.01	6.11	0.1	72.91	1.27
	2-CT	8.56	0.45	1.52	0.32	11.66	1.42	1.37	0.06	6.37	0.27	75.94	3.3
	3-TT	8.43	0.35	1.71	0.26	10.31	1.09	1.44	0.04	6.08	0.21	72.43	2.53
**LEP**	1-CC	8.85	0.23	L. o.		11.56 ^d^	0.73	1.41	0.02	6.2	0.15	74.05	1.78
	2-CT	0.99	0.16	L. o.		10.6	0.55	1.41	0.02	6.09	0.11	72.67	1.38
	3-TT	8.56	0.24	L. o.		9.94 ^d^	0.75	1.38	0.03	6.13	0.15	73.09	1.82
**SCD1**	1-AA	8.96	0.19	2.02	0.13	10.41	0.69	L. o.		L. o.		70.96 ^bc^	1.49
	2-VA	8.85	0.16	1.83	0.1	10.89	0.6	L. o.		L. o.		74.01 ^c^	1.27
	3-VV	8.51	0.47	1.93	0.33	10.61	1.46	L. o.		L. o.		77.93 ^b^	3.26
**SREBF11**	1-LL	8.93 ^a^	0.12	1.91	0.08	10.73	0.54	1.41	0.01	6.14	0.1	73.25	1.26
	2-LS	8.36 ^a^	0.36	1.8	24	10.42	1.01	1.4	0.04	6	0.19	71.58	2.35
**TG**	1-CC	8.84	0.14	1.90 ^a^	0.09	11.04	0.55	1.41	0.02	6.12	0.11	72.99	1.35
	2-CT	8.93	0.23	1.80 ^b^	0.16	9.95	0.74	1.39	0.03	6.19	0.15	73.83	1.79
	3-TT	9.09	0.49	2.61 ^ab^	0.36	9.08	1.43	1.47	0.06	5.93	0.28	70.58	3.42

LSM-last square means; SE-standard error; LWG-live weight gain; DM_LWG-dry matter live weight gain; ME_LWG-metabolic energy live weight gain; LEP-leptin, DGAT1-diacylglycerol O-acyltransferase; SCD1-Stearoyl-CoA desaturase; SREBF11-sterol regulatory element binding transcription factor; TG-thyroglobulin; L. o.-low observation frequency; values with the same letters in column differ significantly: upper case at *p* ≤ 0.01; small case at *p* ≤ 0.05. ^1^ EUROP system; ^2^ on a scale 1 = lean and 5 = well-marbled; ^3^ % of Right hindquarter; ^4^ kg/d; ^5^ Mcal/kg DM; ^6^ MJ/kg gain.

**Table 5:** Effect of breed, rearing intensity, and chosen gene markers on beef quality traits in HF and crossbred steers.

Effect		Wlthaw7		pH 7		L7		a7		b7		WIthaw14		pH 14		L14		a14		b14	
		LSM	Se	LSM	Se	LSM	Se	LSM	Se	LSM	Se	LSM	Se	LSM	Se	LSM	Se	LSM	Se	LSM	Se
**Breed**	1-dairy	4.98 ^a^	0.25	5.4	0.02	34.62 ^A^	1.05	21.22 ^A^	0.5	9.35 ^A^	0.39	5.3	0.31	5.4	0.03	38.3 ^a^	1.32	22.66 ^a^	0.4	10.83 ^A^	0.32
	2-cross	5.70 ^a^	0.26	5.4	0.03	39.48 ^A^	1.09	23.67 ^A^	0.5	11.54 ^A^	0.4	5.9	0.32	5.4	0.03	41.32 ^a^	1.37	23.60 ^a^	0.42	11.97 ^A^	0.33
**Intensity**	1-low	6.47	1.27	5.66 ^a^	0.12	35.26	5.38	23.44	2.6	10.3	1.98	9.75 ^A^	1.56	5.5	0.15	42.9	6.72	27.05 ^b^	2.04	13.7	1.67
	2-high	4.2	1.19	5.17 ^a^	0.12	38.84	5.04	21.43	2.4	10.6	1.85	1.53 ^A^	1.46	5.4	0.14	36.72	6.3	19.21 ^b^	1.91	9.12	1.51
**DGAT1**	1-CC	4.84 ^a^	0.15	5.5	0.01	34.27 ^B^	0.62	21.98	0.3	10.2	0.23	5.2	0.18	5.5	0.02	35.32 ^AB^	0.77	22.71 ^c^	0.23	11.09 ^a^	0.19
	2-CT	5.5	0.49	5.4	0.05	41.59 ^Ba^	2.06	23.49	1	10.6	0.76	5.8	0.6	5.4	0.06	42.70 ^A^	2.57	22.6	0.78	10.9	0.62
	3-TT	5.67 ^a^	0.39	5.4	0.04	35.3 ^a^	1.66	21.86	0.8	10.5	0.61	5.9	0.48	5.4	0.05	41.41 ^B^	2.08	24.04 ^c^	0.63	12.18 ^a^	0.05
**TG**	1-CC	5	0.15	5.4	0.01	35.50 ^a^	0.66	22.39 ^B^	0.3	10.48 ^B^	0.21	5.43 ^a^	0.18	5.4	0.02	36.92	0.86	22.8	0.24	11.2	0.19
	2-CT	4.83	0.28	5.5	0.03	32.83 ^a^	1.21	20.91 ^B^	0.5	9.20 ^B^	0.39	5.3	0.33	5.5	0.03	35.55	1.58	23.1	0.45	11	0.36
	3-TT	4.99	0.57	5.4	0.05	34.81	2.44	21.86	1.1	10.4	0.8	3.98 ^a^	0.67	5.5	0.06	34.11	3.2	22.3	0.91	10.8	0.72
**LEPTIN**	1-CC	5.29	0.26	5.4	0.02	35.67	1.14	21.19 ^a^	0.5	9.59	0.38	5.7	0.31	5.38 ^Aa^	0.03	37.54	1.48	22.2	0.41	10.8	0.33
	2-CT	4.84	0.17	5.5	0.02	34.38	0.73	22.34 ^a^	3.2	10.4	0.24	5.2	0.2	5.48 ^A^	0.02	36.1	0.95	23	0.26	11.3	0.21
	3-TT	5.04	0.29	5.4	0.03	36.41	1.26	22.43	0.6	10.6	0.42	5.2	0.34	5.48 ^a^	0.03	36.68	1.63	22.7	0.45	11.2	0.36
**SCD1**	1-AA	5.07	0.21	5.49 ^Ab^	0.02	33.49 ^a^	0.9	22.49	0.4	10.6	0.31	5.2	0.25	5.50 ^a^	0.02	34.44 ^a^	1.14	22.8	0.33	11.2	0.26
	2-VA	4.9	0.17	5.42 ^A^	0.01	35.98 ^a^	0.71	21.92	0.3	10.1	0.25	5.4	0.2	5.43 ^a^	0.02	37.80 ^a^	0.91	22.9	0.26	11.2	0.21
	3-VV	5.34	0.53	5.38 ^b^	0.05	33.43 ^a^	2.27	21.4	1	9.61	0.79	6	0.64	5.5	0.06	34.72	2.88	21.5	0.83	9.99	0.66
**SREBF11**	1-LL	4.95	0.14	5.4	0.01	34.93	0.64	22.1	0.3	10.2	0.21	5.3	0.17	5.5	0.02	36.2	0.08	22.8	0.23	11.2	0.18
	2-LS	5.22	0.38	5.5	0.04	35.77	1.67	22.21	0.8	10.9	0.56	5.2	0.46	5.5	0.04	39.18	2.1	23.2	0.6	11.3	0.48
**AgeSlaug**	regression											**						*			

LSM-last square means; SE-standard error; Wlthaw7-drip loss (%) after 7 days; pH 7-pH after 7 days; L7-meat brightness after 7 days; a7-meat red-color saturation after 7 days; b7-meat yellow-color saturation after 7 days; Wlthaw14-drip loss (%) after 14 days; pH 14-pH after 14 days; L14-meat brightness after 14 days; a14-meat red-color saturation after 14 days; b14-meat yellow-color saturation after 14 days; LEP-leptin, DGAT1-diacylglycerol O-acyltransferase; SCD1-Stearoyl-CoA desaturase; SREBF11-sterol regulatory element binding transcription factor; TG-thyroglobulin; AgeSlaug-age at slaughter; values with the same letters differ significantly: upper case at *p* ≤ 0.01; small case at *p* ≤ 0.05. ** significant at a level of 0.01; * significant at a level of 0.05.

## Data Availability

All data generated or analyzed during the study are included in this published article. The datasets used and/or analyzed in the current study are available from the corresponding author on reasonable request.

## Supplementary Materials

The following supporting information can be downloaded at: https://www.mdpi.com/article/10.3390/genes15070843/s1, Table S1: PCR and digestion conditions; Table S2: Effect of breed, rearing intensity, and the chosen gene polymorphisms on beef sensory quality traits in HF and crossbred steers.
