# Supplementary material for: Genetic Markers Related to Meat Quality Properties in Fattened HF and HF x Charolaise Steers
Source: Genes (Basel). 2024 Jun 27;15(7):843. doi: 10.3390/genes15070843 (PMC11276422; doi:10.3390/genes15070843)
Supplement: Supplementary file 1 [file genes-15-00843-s001.zip › genes-3011875-supplementary.pdf]

Supplement S1. PCR and digestion conditions

Gene	PCR mixture	PCR condition		Restriction enzyme	Digestion condition	Reference
SCD1	Mix 10 μl<br>PF 1 μl<br>PR 1 μl<br>H_20 6 μl<br>DNA 2 μl<br>Total 20 μl	1) 95 °C - 5 min<br>2) 95 °C - 30s<br>3) 60 °C - 30s<br>4) 72 °C - 45s<br>5) 72 °C - 7 min<br>6) 4 °C - ∞	35 cycles (steps 2–4)	Fnu4HI	Reaction condition 37 °C, 15 min<br>Digestion protocol:<br>Enzyme 1 μl<br>DNA 10 μl<br>10x buffer 5 μl<br>H_2O 34 μl<br>Total 50 μl<br><br>Enzyme inactivation on ice	[10]
LEP	Mix 10 μl<br>PF 1 μl<br>PR 1 μl<br>H_20 6 μl<br>DNA 2 μl<br>Total 20 μl	1) 94 °C - 2 min<br>2) 94 °C - 45s<br>3) 52 °C - 45s<br>4) 72 °C - 55s<br>5) 72 °C - 3 min<br>6) 4 °C - ∞	35 cycles (steps 2–4)	BspEI	Reaction condition 37 °C, 15 min<br>Digestion protocol:<br>Enzyme 1 μl<br>DNA 10 μl<br>10x buffer 5 μl<br>H_2O 34 μl<br>Total 50 μl<br><br>Enzyme inactivation at 80 °C, 20 min then on ice	[19]
TG	Mix 10 μl<br>PF 1 μl<br>PR 1 μl<br>H_20 6 μl<br>DNA 2 μl<br>Total 20 μl	1) 95 °C - 10 min<br>2) 94 °C - 30s<br>3) 52 °C - 30s<br>4) 72 °C - 60s<br>5) 72 °C - 7 min<br>6) 4 °C - ∞	35 cycles (steps 2–4)	BstYI	Reaction condition 60 °C, 15 min<br><br>Digestion protocol:<br>Enzyme 1 μl<br>DNA 10 μl<br>10x buffer 5 μl<br>H_2O 34 μl<br>Total 50 μl<br><br>Enzyme inactivation on ice	[20]
DGAT1	Mix 10 μl<br>PF 1 μl<br>PR 1 μl<br>H_20 6 μl<br>DNA 2 μl<br>Total 20 μl	1) 95 °C - 10 min<br>2) 94 °C - 30s<br>3) 52 °C - 30s<br>4) 72 °C - 60s<br>5) 72 °C - 7 min<br>6) 4 °C - ∞	35 cycles (steps 2–4)	CfrI	Reaction condition 37 °C, 60 min<br><br>Digestion protocol:<br>Enzyme 1 μl<br>DNA 10 μl<br>10x buffer 5 μl<br>H_2O 34 μl<br>Total 50 μl<br><br>Enzyme inactivation at 65 °C, 10 min then on ice	[20]
SREBF11	Mix 10 μl<br>PF 1 μl<br>PR 1 μl<br>H_20 6 μl<br>DNA 2 μl<br>Total 20 μl	1) 95 °C - 5 min<br>2) 95 °C - 30s<br>3) 60 °C - 30s<br>4) 72 °C - 45s<br>5) 72 °C - 7 min<br>6) 4 °C - ∞	35 cycles (steps 2–4)	-	-	[10]

Supplement S2. Effect of breed, rearing intensity, and the chosen gene polymorphisms on beef sensory quality traits in HF and crossbred steers.

Effect		Meat color - brown		Bright meat color		Shear force		Juiciness		Tenderness		Taste - total meat taste		Taste - sweet		Taste - sour		Taste - umami		Taste - metallic	
		LSM	Se	LSM	Se	LSM	Se	LSM	Se	LSM	Se	LSM	Se	LSM	Se	LSM	Se	LSM	Se	LSM	Se
**Breed**	1 - dairy	26.5[a]	1.6	45.14[A]	2.37	41.28[a]	2.96	45.8	2.61	53.1	3.88	43.98	0.94	23.56	0.65	23.7	0.75	32.36	0.8	34.9	0.68
	2 - cross	20.9[a]	1.91	36.61[A]	2.83	51.76[a]	3.53	47.3	3.11	44.5	4.63	43.15	1.12	23.55	0.77	24.8	0.89	32.73	0.95	33.68	0.82
**Intensity**	1 - low	21.91	9.39	35.71	13.9	55.33	17.3	55.7	15.3	54.4	22.7	49.46	5.48	26.12	3.79	26.4	4.39	35.03	4.65	36.2	4.0
	2 - high	25.53	9.12	52.03	13.5	37.71	16.8	37.4	14.8	43.2	22.1	37.68	5.32	20.98	3.68	22.1	4.26	30.06	4.52	32.38	3.89
**DGAT1**	1 - CC	22.98	1.17	40.99	1.73	47.39	2.16	44.2	1.9	45.9	2.83	42.86	0.68	23.48	0.47	24.3	0.55	31.58	0.58	34.88	0.5
	2 - CT	26.1	3.36	41.4	4.97	39.6	6.2	50.9	5.47	52.4	8.13	45.09	1.96	22.36	1.36	25.1	1.57	34.3	1.67	34.93	1.43
	3 - TT	22.07	2.61	34.21	3.86	52.57	4.82	44.6	4.25	48	6.32	42.75	1.53	24.82	1.06	23.4	1.22	31.76	1.3	33.05	1.11
**TG**	1 - CC	23.55	0.88	40.15	1.37	46.6	1.72	45.3	1.46	47.2	2.14	43.3	0.52	23.45	0.38	24.3	0.42	32.12	0.44	34.65	0.39
	2 - CT	20.67	3.04	42.39	4.73	49.38	5.95	44.2	5.05	45.6	7.39	41.69	1.78	23.52	1.29	24.2	1.44	30.52	1.54	35.13	1.36
**LEP**	1 - CC	23.27	1.37	41.96	2.11	48.37	2.63	44	2.27	44.3	3.28	42.74	76	23.07	0.55	24.4	0.66	31.64	0.7	35.27	0.6
	2 - CT	24.2	1.35	39.44	2.07	47.11	2.58	46.3	2.23	49.3	3.21	44.23	0.75	24.17	0.54	24.2	0.64	32.42	0.69	34.32	0.59
	3 - TT	21.35	2.3	38.43	3.53	42.05	4.4	45.6	3.8	48.2	5.47	41.57	1.27	22.54	0.92	24.4	1.1	31.87	1.18	34.21	1.0
**SCD1**	1 - AA	20.87	3.2	36.41	4.88	54.53	5.98	43.8	5.31	38.3	7.49	40.4	1.74	23.48	1.23	22.4	1.4	29.2	1.48	34.38	1.38
	2 - VA	23.72	0.97	40.61	1.47	46.02	1.81	45.4	1.6	48	2.26	43.64	0.53	23.59[a]	0.37	24.5	0.42	32.44	0.45	34.63	0.42
	3 - VV	21.13	4.93	44.81	7.51	41.48	9.21	45	8.18	51.7	11.5	39.51	2.69	19.35[a]	1.9	26.9	2.15	28.98	2.29	37.36	2.12
**SREBF11**	1 - LL	23.61	0.91	40.83	1.39	47.47	1.74	44.7	1.47	46	2.12	43.09	0.54	23.35	0.38	24.4	0.43	32	0.47	34.76	0.4
	2 - LS	21.55	2.34	36.59	3.55	41.99	4.46	49.1	3.78	54.5	5.43	43.87	1.38	24.21	0.98	23.5	1.09	32.1	1.2	34.19	1.04
**LW SLAUGH**	regression																				

**LW START**	regression	**	
**AGE START**	regression	*	

LSM - last square means; **SE** - standard error; **LEP** - leptin; **DGAT1** - diacylglycerol O-acyltransferase; **SCD1** - Stearoyl-CoA desaturase; **SREBF11** - sterol regulatory element binding transcription factor; **TG** - thyroglobulin; the values of the least square means (LSM) with the same letters differ significantly: upper case at $p \leq 0.01$; small case at $p \leq 0.05$; ** significant at a level of 0.01; * significant at a level of 0.05
